# Correlation Between Prognostic Biomarker *SLC1A5* and Immune Infiltrates in Various Types of Cancers Including Hepatocellular Carcinoma

**DOI:** 10.3389/fonc.2021.608641

**Published:** 2021-07-22

**Authors:** Junsheng Zhao, Zhongli Yang, Mingmin Tu, Wei Meng, Hainv Gao, Ming D. Li, Lanjuan Li

**Affiliations:** ^1^ State Key Laboratory for Diagnosis and Treatment of Infectious Diseases, National Clinical Research Center for Infectious Diseases, Collaborative Innovation Center for Diagnosis and Treatment of Infectious Diseases, The First Affiliated Hospital, Zhejiang University School of Medicine, Hangzhou, China; ^2^ Department of Clinical Laboratory, Hangzhou Tongchuang Medical Laboratory, Hangzhou, China; ^3^ Department of Clinical Laboratory, Zoucheng People’s Hospital, Zoucheng, China; ^4^ Department of Infectious Diseases, ShuLan (Hangzhou) Hospital Affiliated to Zhejiang Shuren University Shulan International Medical College, Hangzhou, China; ^5^ Research Center for Air Pollution and Health, Zhejiang University, Hangzhou, China

**Keywords:** glutamine, hepatocellular carcinoma, immune cell infiltration, lower-grade glioma, prognosis determination, *SLC1A5*

## Abstract

**Background:**

Solute carrier family 1 member 5 (*SLC1A5*) is a major glutamine transporter and plays a key role in tumor growth. The main objectives of this study were to visualize the prognostic landscape of *SLC1A5* in multiple cancers and determine the relations between *SLC1A5* expression and tumor immunity.

**Methods:**

*SLC1A5* expression and its effect on tumor prognosis were analyzed using multiple online tools Oncomine, Gene Expression Profiling Interactive Analysis, PrognoScan, and Kaplan-Meier plotter with their own datasets as well as the data from The Cancer Genome Atlas. The correlations between *SLC1A5* and tumor immune infiltrates were determined *via* TIMER.

**Results:**

*SLC1A5* expression was significantly higher in several types of cancers, including hepatocellular carcinoma (HCC), compared with corresponding normal tissues. High *SLC1A5* expression correlated with poor overall survival and with disease-free survival related to alcohol consumption. Moreover, *SLC1A5* expression correlated positively with the numbers of tumor-infiltrating B cells, CD4^+^ T and CD8^+^ T cells, macrophages, neutrophils, and dendritic cells in HCC and in lower-grade glioma (LGG). Also, *SLC1A5* expression showed strong correlations with diverse immune marker sets in HCC and LGG, indicating its role in regulating tumor immunity.

**Conclusions:**

*SLC1A5* represents a useful prognostic biomarker in multiple cancers, and its expression correlates highly with tumor immune-cell infiltration, especially in HCC and LGG.

## Introduction

Hepatocellular carcinoma (HCC) is one of the most common malignancies and ranks third among the causes of cancer-related deaths worldwide, with a 5-year survival rate of < 5% (1, 2). The majority of cases are caused by virus infections, non-alcoholic steatohepatitis, or alcohol-related liver diseases (3–5). Commonly, liver cancer is detected in late stages and thus is difficult to treat, and it does not respond well to traditional chemotherapy. Immune checkpoint therapy has been developed as a new method for treating various cancers, including HCC (6). During the past several years, immunotherapy has achieved impressive results, with promising prospects for immune checkpoint inhibitors (ICIs) in patients with different cancers, including HCC (7–10).

However, only a limited proportion of patients respond well to current immunotherapies (11–13). Recently, high infiltration of immune cells, such as natural killer (NK) cells and CD8^+^ and CD4^+^ T effector cells, was reported to be associated with a response to clinical anti-PD1/PD-L1-based therapy (14, 15). Some immune cells in the tumor microenvironment (TME), including dendritic cells (DC), CD8^+^ and CD4^+^ T lymphocytes, and NK cells, also play an important role in cancer initiation and progression (16). Thus, there is an urgent need for the elucidation of the molecular mechanisms of cancer immunology including HCC and development of new immune-related therapies for different types of cancers.

Glutamine is a conditionally essential nutrient for rapidly proliferating tumor cells, as glutaminolysis is critical for activation of the mammalian target of the rapamycin complex 1 (mTORC1) nutrient-signaling pathway, which regulates cell growth and protein translation in cancer cells. Recent research indicates that glutamine uptake influences specific immune cell infiltration in breast cancer (17). Also, JHU083 (a glutamine antagonist) can suppress the growth of MYC-driven medulloblastoma (18) and improves the antitumor effects of anti-PD1 therapy in a mouse model (MC38) (19). These studies indicate a plausible prospect for using a combination of a glutamine inhibitor and immune-checkpoint inhibitor to treat cancers.

Solute carrier family 1 member 5 (SLC1A5), also known as alanine–serine-cysteine transporter 2 (ASCT2), is a cell-surface solute-carrying transporter that mediates uptake of neutral amino acids including glutamine and acts as one of the key amino acid transporters. It has received great attention because of its role in supporting tumor metabolism (20). Elevated *SLC1A5* expression has been linked to poor survival in many human cancers, including those of the liver (21), lung (22), breast (23), colon (24), and head and neck squamous (25). Although inhibition of SLC1A5 failed to affect HCC proliferation (26), a variant of SLC1A5 was found to be a gatekeeper for glutamine metabolism and metabolic reprogramming in cancer cells (27). Elucidation of the correlation of *SLC1A5* with tumor immune-cell infiltration might help in understanding the mechanisms of response and resistance to the respective inhibitors, ultimately paving the way for development of predictive biomarkers and novel treatments.

We performed a systematic analysis of the correlation of *SLC1A5* expression with the prognosis of cancer patients attested to in several databases, including Oncomine, PrognoScan, Gene Expression Profiling Interactive Analysis (GEPIA), and Kaplan-Meier plotter. Furthermore, we explored the correlation of *SLC1A5* expression with immune infiltration *via* the Tumor Immune Estimation Resource (TIMER) and GEPIA databases. Our findings showed the prognostic value of *SLC1A5* in HCC and lower-grade glioma (LGG) and provided novel insights into the correlation of and mechanism active between *SLC1A5* expression and tumor immunity.

## Materials and Methods

### *SLC1A5* Expression Analysis

The mRNA concentrations of *SLC1A5* in diverse types of cancers were identified from the Oncomine database (https://www.oncomine.org/resource/login.html) (28). The threshold for significance was determined according to the following values: p < 0.001, fold change > or = 1.5, and gene ranking of all.

The level 4 gene-expression data (FPKM normalized) of TCGA were downloaded from the UCSC Xena browser (https://gdc.xenahubs.net) (29). The *SLC1A5* expression in 24 types of cancers for which expression data from adjacent normal controls were available were first log_2_(TPM+1)-transformed and then used to assess expression difference by using the Wilcoxon test. The *SLC1A5* expression difference between tumor and normal tissues in LGG, ovarian carcinoma (OV), testicular germ cell tumors (TGCT), and uterine carcinosarcoma (UCS) were analyzed in GEPIA (30).

### Survival Analysis

The Kaplan-Meier method was used to assess the correlation of *SLC1A5* expression and the overall survival rate in 33 types of cancers using 7,489 cancer samples from TCGA. With multivariate Cox analysis, we evaluated the influence of *SLC1A5* expression and other clinical factors on OS in both LGG and HCC. A p value < 0.05 was set as the threshold for significance. The time-dependent receiver operating characteristic (ROC) curve was used to estimate the predictive performance of *SLC1A5* expression in LGG and HCC.

Additionally, the correlation between *SLC1A5* expression and survival in pan-cancer was analyzed by PrognoScan (http://www.prognoscan.org/) (31). The *SLC1A5* expression was searched for all available microarray datasets in PrognoScan to determine its relation to prognosis, such as OS, DSS, relapse-free survival (RFS), and distant metastasis-free survival (DMFS) using Cox p value < 0.05 as the threshold for significance.

Kaplan-Meier plotter (http://kmplot.com/analysis/) was used to analyze the relation between *SLC1A5* expression and survival days in liver cancer with different clinicopathologic factors (32), which were assessed by hazard ratio (HR) with 95% confidence intervals and log-rank p values.

### Correlations Between *SLC1A5* Expression and Immune Cells in TIMER and GEPIA

The correlation between *SLC1A5* expression and the abundance of tumor-infiltrating immune cells (TIICs), including CD4^+^ cells, CD8^+^ cells, B cells, neutrophils, macrophages, and DC was analyzed using the TIMER web tool (https://cistrome.shinyapps.io/timer/) (33). The relation between *SLC1A5* expression and tumor purity was also determined (34). In addition, the correlation between *SLC1A5* expression and gene markers of TIICs, including B cells, CD8^+^ cells, follicular helper T cells (Tfh), T-helper 1 (Th1) cells, T-helper 2 (Th2) cells, T-helper 9 (Th9) cells, T-helper 17 (Th17) cells, T-helper 22 (Th22) cells, regulatory T cells (Tregs), exhausted T cells, M1 macrophages, M2 macrophages, tumor-associated macrophages (TAM), monocytes, NK cells, neutrophils, and DCs also was explored in LGG and HCC *via* correlation modules.

### Gene Ontology (GO) Enrichment Analysis

Gene set enrichment analysis (GSEA) was performed using normalized gene expression data obtained from TCGA with the R packages clusterProfiler, gerichplot, and ggplot2 (35). The Broad Molecular Signatures Database (MSigDB v 7.1) set C5 was used, which contain genes annotated by the same GO term. Enriched gene sets in *SLC1A5* higher expression group were identified using 1,000 permutations of the phenotype labels. Terms with a p value < 0.05 were considered significantly enriched.

### Statistical Analysis

All statistical analyses were conducted using R software (Version 3.6.2). The overall survival curves were estimated by the Kaplan-Meier method, and the differences between survival distributions were assessed with the two-sided log-rank test as implemented in the R package survival. A modified drawing survival curve function ‘ggsurvplot,’ as implemented in the R package survminer, was used to draw Kaplan-Meier survival curves. The correlation of gene expression with the measure of interest was evaluated by Spearman’s correlation, and the strength of the correlation was determined using the following criteria for the absolute value: 0.00–0.19 = very weak; 0.20–0.39 = weak; 0.40–0.59 = moderate; 0.60–0.79 = strong; and 0.80–1.0 = very strong. For the comparison of two groups, statistical significance for non-normally distributed variables was analyzed using the Wilcoxon rank-sum test. A p value < 0.05 was considered statistically significant.

## Results

### *SLC1A5* mRNA Expression in Human Cancers

**Figure 1:**
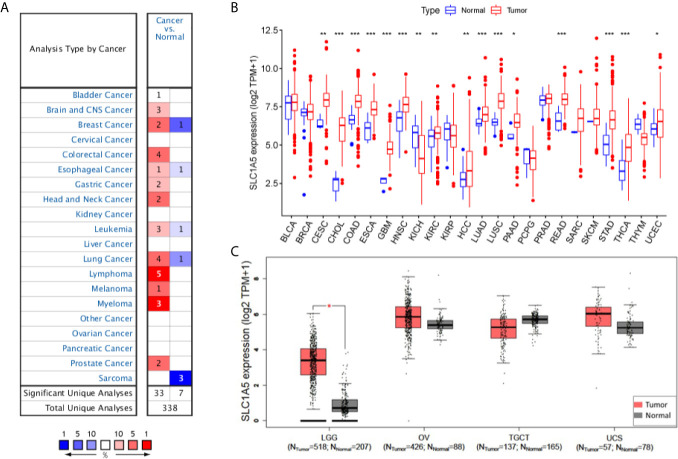
*SLC1A5* expression in cancers. **(A)** Altered expression in different cancer tissues compared with normal tissues in ONCOMINE. Number in each cell is the number of datasets. **(B)** Expression in different solid tumor types from TCGA database. *p < 0.05, **p < 0.01, ***p < 0.001. **(C)** Expression in four complementary solid tumor types from TCGA and GTEx projects in GEPIA.

The *SLC1A5* mRNA concentrations in different tumors and normal tissues linked to multiple cancer types were analyzed in Oncomine. The *SLC1A5* mRNA concentrations were significantly higher in the central nervous system (CNS), breast, colorectum, esophagus, stomach, head and neck, leukemia, lung, lymphoma, melanoma, myeloma, and sarcoma tissues compared with the corresponding normal tissues (**Figure 1A** and **Supplementary Table 1**). In addition, lower expression of *SLC1A5* was observed in breast, esophagus, leukemia, lung, and sarcoma tumors in some datasets.

To determine *SLC1A5* expression in cancer in more detail, we examined expression in pan-cancers based on the TCGA database. Under the cutoff value of p < 0.05, no tumor showed significantly lower *SLC1A5* expression than its corresponding normal tissue. Meanwhile, *SLC1A5* was significantly more abundant in 16 types of tumor tissues, namely cervical squamous-cell carcinoma and endocervical adenocarcinoma (CESC), cholangiocarcinoma (CHOL), colon adenocarcinoma (COAD), esophageal carcinoma (ESCA), glioblastoma multiforme (GBM), head and neck squamous-cell carcinoma (HNSE), kidney chromophobe cancer (KICH), renal clear-cell carcinoma (KIRC), HCC, lung adenocarcinoma (LUAD), lung squamous-cell carcinoma (LUSC), pancreatic adenocarcinoma (PAAD), rectal adenocarcinoma (READ), stomach adenocarcinoma (STAD), thyroid carcinoma (THCA), and uterine corpus endometrial carcinoma (UCEC) than in their corresponding normal tissues (**Figure 1B**). As shown in **Figure 1C**, *SLC1A5* was significantly overexpressed in LGG but not in OV, TGCT, or UCS.

### Prognostic Potential of *SLC1A5* in Cancers

**Figure 2:**
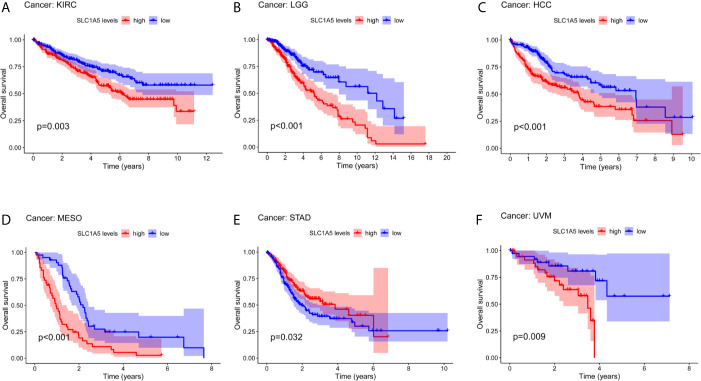
Overall survival (OS) curves comparing high and low expression of *SLC1A5* kidney clear-cell carcinoma (KIRC) **(A)**; lower-grade glioma (LGG) **(B)**; hepatocellular carcinoma (HCC) **(C)**; mesothelioma (MESO) **(D)**; stomach adenocarcinoma (STAD) **(E)**, and uveal melanoma (UVM) **(F)** in TCGA database.

Next, we investigated the prognostic value of *SLC1A5* for pan-cancer recorded in different databases. We used gene expression data from TCGA to assess *SLC1A5*-related OS in 33 types of cancers with the Kaplan-Meier method. We revealed *SLC1A5* to be an adverse prognostic factor in KIRC (p = 0.003), LGG (p < 0.001), HCC (p < 0.001), MESO (p < 0.001), and UVM (p = 0.009) (**Figure 2**). Conversely, *SLC1A5* had a marginally protective role in STAD (p = 0.032) (**Figure 2E**).

**Figure 3:**
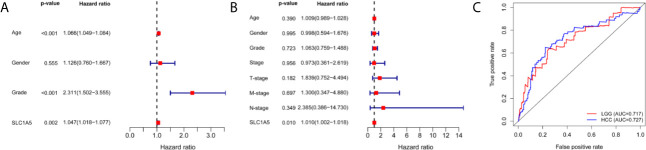
Multivariate Cox analysis of *SLC1A5* expression and other clinicopathological variables in LGG **(A)** and HCC **(B)**. **(C)** ROC curve of differential *SLC1A5* expression in LGG and HCC. OS, overall survival; LGG, lower-grade glioma; HCC, hepatocellular carcinoma; and ROC, receiver operating characteristic.

As shown in **Figures 3A, B**, our multivariate analyses revealed that *SLC1A5* expression was an independent factor for prognosis (p < 0.05). *SLC1A5* expression showed promising prognostic power, as the AUC for predicting overall survival was 0.727 and 0.717 in LGG and HCC (**Figure 3C**).

In PrognoScan, we explored the relation between *SLC1A5* expression and the prognosis of each type of cancer. Notably, highly expressed *SLC1A5* correlated significantly with poor OS in five cancer types, namely bladder (HR = 1.51; 95% CI 1.04, 2.19; p = 0.0315), blood (HR = 1.69; 95% CI 1.05, 2.73; p = 0.0317), brain (HR = 1.96; 95% CI 1.20, 3.19; p = 0.0069), breast (HR = 1.39; 95% CI 1.15, 1.68; p = 5.9e-5), and skin (HR = 3.87; 95% CI 1.34, 11.14; p = 0.0122). In some datasets, high *SLC1A5* expression correlated with a better prognosis in breast (DMFS HR = 0.64; 95% CI 0.42, 0.94; p = 0.0363) and ovarian (OS HR = 0.66; 95% CI 0.47, 0.93; p = 0.0181) (**Supplementary Table 2**) lesions.

Using the Kaplan-Meier plotter database, we further assessed the prognostic value of *SLC1A5* expression in breast, ovarian, lung, and gastric cancer. High *SLC1A5* expression was significantly associated with a poor prognosis in breast (OS HR = 1.4; 95% CI 1.13, 1.74; p = 0.002), gastric (OS HR = 1.88; 95% CI 1.58, 2.24; p = 2.7e-13), and lung (OS HR = 1.51; 95% CI 1.33, 1.72; p = 1.6e-10) (**Supplementary Figures 1A–G**). Meanwhile, high *SLC1A5* expression was significantly associated with a better prognosis in ovarian cancer (OS HR = 0.85; 95% CI 0.74, 0.96; p = 0.011) (**Supplementary Figure 1H**).

### Relation Between *SLC1A5* Expression and Clinicopathology in HCC

To understand better the relevance and underlying mechanisms of *SLC1A5* expression in cancers, we assessed the relation between *SLC1A5* expression and the clinical characteristics of HCC patients in the Kaplan-Meier plotter database. High *SLC1A5* expression correlated with both poor OS (HR = 2.68; p = 0.0035) and PFS (HR = 1.83; p = 0.021) in alcohol consumers (**Table 1**). Elevated *SLC1A5* expression correlated significantly with worse OS in HCC patients with the following clinical characteristics: male, stage 3, grade 2, AJCC_T3 and microvascular invasion, Asian, no alcohol consumption, and without hepatitis virus infection (p < 0.05). These results demonstrated the prognostic significance of *SLC1A5* expression in HCC patients based on their clinical characteristics, especially for patients with advanced stages of cancer.

**Table 1:** Correlation of *SLC1A5* mRNA expression and prognosis in hepatocellular carcinoma (HCC) with different clinicopathological factors in Kaplan-Meier plotter.

Clinicopathological factors	Overall survival			Progression-free survival		
	Sample size (n)	Hazard ratio (HR)	p value	Sample size (n)	Hazard ratio (HR)	p value
Sex						
Female	118	1.31 (0.75–2.28)	0.34	121	0.83 (0.5–1.39)	0.48
Male	246	2.61 (1.63–4.18)	**3.7e-05**	249	1.38 (0.97–1.98)	0.076
Tumor stage						
1	170	1.3 (0.71–2.39)	0.4	171	1.01 (0.61–1.65)	0.98
2	83	1.88 (0.84–4.23)	0.12	85	0.69 (0.37–1.3)	0.25
1+2	253	1.5 (0.92–2.43)	0.1	266	1 (0.69–1.46)	1
3	83	2.37 (1.28-4.38)	**0.0048**	85	1.22 (0.71–2.11)	0.47
2+3	166	2.26 (1.4–3.67)	**0.00067**	170	1.25 (0.84–1.86)	0.28
4	5	–	–	5	–	–
3+4	87	2.17 (1.2–3.91)	**0.0089**	90	1.21 (0.72–2.06)	0.47
Tumor grade						
1	55	1.48 (0.58–3.75)	0.41	55	1.5 (0.69–3.25)	0.3
2	174	2.32 (1.36–3.99)	**0.0016**	177	1.1 (0.71–1.7)	0.67
3	118	1.46 (0.8–2.67)	0.22	121	0.91 (0.55–1.5)	0.71
4	12	–	–	12	–	–
AJCC_T (American Joint Committee on Cancer T) stage						
1	180	1.38 (0.77–2.47)	0.28	181	1.12 (0.69–1.81)	0.64
2	90	1.95 (0.91–4.17)	0.08	93	0.97 (0.55–1.72)	0.92
3	78	2.59 (1.37–4.9)	**0.0024**	80	1.33 (0.75–2.34)	0.33
4	13	–	–	13	–	–
Vascular invasion						
Micro	90	4.44 (1.67–11.81)	**0.0011**	92	0.69 (0.38–1.25)	0.21
None	203	1.41 (0.85–2.36)	0.19	205	1.28 (0.82–1.99)	0.28
Macro	16	–	–	16	–	–
Race						
White	181	1.35 (0.85–2.12)	0.2	184	1.18 (0.8–1.75)	0.41
Asian	155	3.46(1.78–6.74)	**9.8e-05**	157	1.5 (0.93–2.4)	0.092
Alcohol consumption						
Yes	115	2.68 (1.35–5.33)	**0.0035**	117	1.83 (1.09–3.09)	**0.021**
None	202	1.63 (1.03–2.59)	**0.037**	205	1.02 (0.68–1.53)	0.91
Hepatitis virus infection						
Yes	150	1.48 (0.77–2.85)	0.23	153	1.1 (0.7–1.75)	0.67
None	167	2.15 (1.35–3.41)	**0.00094**	169	1.24 (0.8–1.92)	0.33

Bold values indicate P < 0.05.

### Positive Correlation of *SLC1A5* Expression and Immune Infiltration in HCC

Considering that the previous study had implicated tumor-infiltrating lymphocytes as independent predictors of sentinel lymph node status and survival in cancer patients (36), we assessed the relation between *SLC1A5* expression and infiltrating immune cells in 39 types of cancers including HCC using the TIMER database. The extent of *SLC1A5* expression correlated significantly with tumor purity in 20 types of cancer. In addition, *SLC1A5* mRNA correlated significantly with the extent of infiltration of B cells in 14 cancer types, CD4^+^ cells in 15 cancer types, CD8^+^ cells in 12 cancer types, DC in 20 cancer types, macrophages in 17 cancer types, and neutrophils in 17 cancer types (**Supplementary Table 3**).

**Figure 4:**
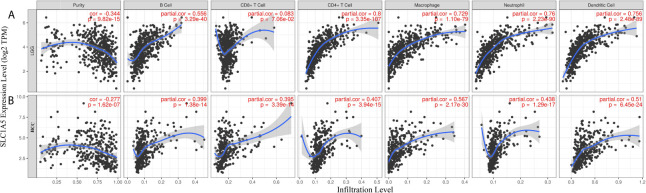
Correlation of *SLC1A5* expression with immune infiltration in lower-grade glioma (LGG) and hepatocellular carcinoma (HCC). **(A)** Expression was significantly negatively correlated with tumor purity and had significant positive correlations with numbers of infiltrating B cells, CD4^+^ cells, macrophages, neutrophils, and DC in LGG but not CD8^+^ cells (n = 516). **(B)** Expression was significantly negatively related to tumor purity and had significant positive correlations with numbers of infiltrating B cells, CD8^+^ cells, CD4^+^ cells, macrophages, neutrophils, and DC in HCC (n = 371).

We further observed that high *SLC1A5* expression correlated with abundant infiltration of immune cell types in LGG and HCC (**Figure 4**). For LGG, *SLC1A5* expression had significantly positive correlations with the extent of infiltration of B cells (r = 0.556; p = 3.29e-40), CD4^+^ cells (r = 0.8; p = 3.35e-107), macrophages (r = 0.729; p = 1.10e-79), neutrophils (r = 0.76; p = 2.23e-90), and DC (r = 0.756; p = 2.48e-89). In HCC, high expression of *SLC1A5* correlated positively with the extent of infiltration of B cells (r = 0.399; p = 1.38e-14), CD8^+^ cells (r = 0.395; p = 3.39e-14), CD4^+^ cells (r = 0.407; p = 3.94e-15), macrophages (r = 0.567; p = 2.17e-30), neutrophils (r = 0.438; p = 1.29e-17), and DC (r = 0.51; p = 6.45e-24). These results indicated that *SLC1A5* expression correlated with immune cell infiltration of tissues of LGG and HCC.

### Correlation Analysis Between *SLC1A5* mRNA and Markers of Different Subsets of Immune Cells in HCC

To further investigate the potential relation between *SLC1A5* and infiltrating immune cells, we examined the correlations between *SLC1A5* expression and several immune cell markers in LGG and HCC in the TIMER database. Because the tumor purity of clinical samples influences the analysis of immune infiltration, the correlation analysis was adjusted for purity (**Table 2**). After this adjustment, *SLC1A5* expression correlated significantly with 36 of 45 immune cell markers in HCC and 40 of 45 in LGG (**Table 2**). Only M1 macrophages in HCC and M2 macrophages in LGG revealed no marker correlated with *SLC1A5* expression. These findings confirmed that *SLC1A5* expression correlated with immune cell infiltration in LGG and HCC. The strongest correlations were found between *SLC1A5* with *PU.1* (*SPI1*) and *SLC1A5* and *CD86*. Interestingly, we found a significantly positive correlation between *SLC1A5* expression and prototypical immunotherapy biomarkers, including *PD-1* and *CTLA4* (**Table 2**). These findings suggest that *SLC1A5* could affect the response to immunotherapy.

**Table 2:** Correlation between *SLC1A5* and gene-markers of immune cells in TIMER.

Cell type	Gene marker	HCC (hepatocellular carcinoma)				LGG (lower-grade glioma)			
		None		Purity		None		Purity	
		cor	p	Cor	p	Cor	p	Cor	p
B cell	CD19	0.3398	***	0.3370	***	0.4023	***	0.3910	***
	CD20	0.1637	**	0.1722	**	0.0259	5.58E-01	0.0216	6.37E-01
	CD38	0.3851	***	0.3951	***	0.1664	***	0.1630	***
CD8+ T cell	CD8A	0.3701	***	0.3815	***	0.12125	**	0.1302	**
	CD8B	0.3783	***	0.3835	***	0.1145	**	0.1163	*
Follicular helper T cell (Tfh)	CXCR5	0.3765	***	0.3772	***	0.2644	***	0.2716	***
	ICOS	0.4578	***	0.4644	***	0.3899	***	0.3884	***
	BCL-6	0.1534	**	0.1734	**	0.2740	***	0.2428	***
T helper cell 1	IL12RB2	0.1207	*	0.1390	**	-0.1858	***	-0.1926	***
	WSX-1	0.5819	***	0.5695	***	-0.0460	2.97E-01	-0.0589	1.98E-01
	T-BET	0.2537	***	0.2606	***	0.2326	***	0.2279	***
T helper cell 2	CCR3	0.4105	***	0.4260	***	0.4008	***	0.4158	***
	STAT6	0.1221	*	0.1232	*	0.4297	***	0.4515	***
	GATA-3	0.4627	***	0.4647	***	0.4101	***	0.4170	***
T helper cell 9	TGFBR2	0.1054	*	0.1226	*	0.5478	***	0.5387	***
	IRF4	0.4315	***	0.4326	***	0.2347	***	0.2312	***
	PU.1 (SPI1)	0.6169	***	0.6284	***	0.8552	***	0.8590	***
T helper cell 17	IL-21R	0.5652	***	0.5713	***	0.0375	3.95E-01	0.0372	4.16E-01
	IL-23R	0.2237	***	0.2315	***	0.1557	***	0.1370	**
	STAT3	0.2438	***	0.2600	***	0.4631	***	0.4360	***
T helper cell 22	CCR10	0.4296	***	0.4276	***	0.1560	***	0.1554	***
	AHR	0.0425	4.14E-01	0.0597	2.68E-01	0.3359	***	0.3343	***
Regulatory T cell	FOXP3	0.0804	1.22E-01	0.1106	*	-0.2324	***	-0.2199	***
	CCR8	0.4140	***	0.429576	***	0.1423	**	0.1563	***
	CD25	0.5132	***	0.52958	***	0.240913	***	0.2357	***
T cell exhaustion	PD-1	0.5198	***	0.521879	***	0.499475	***	0.5005	***
	CTLA4	0.5080	***	0.510929	***	0.315704	***	0.3155	***
Macrophage	CD68	0.4707	***	0.467853	***	0.819322	***	0.8244	***
	CD11b	0.4938	***	0.504546	***	0.780011	***	0.7777	***
M1-type (classically activated) macrophage	NOS2	-0.0755	1.47E-01	-0.07327	1.74E-01	-0.14188	**	-0.1469	**
	ROS	-0.0738	1.56E-01	-0.04904	3.63E-01	-0.27808	***	-0.2653	***
M2-type (alternatively activated) macrophage	ARG1	-0.2455	***	-0.22391	***	0.037151	4.00E-01	0.0371	4.18E-01
	MRC1	0.0891	8.65E-02	0.11013	*	0.123821	**	0.1114	*
Tumor-associated-macrophage (TAM)	HLA-G	0.1107	*	0.116115	*	0.364023	***	0.3675	***
	CD80	0.4958	***	0.526274	***	0.468418	***	0.4587	***
	CD86	0.5767	***	0.591055	***	0.839263	***	0.8441	***
Monocyte	CD14	-0.2327	***	-0.21883	***	0.7049	***	0.7050	***
	CD16	0.4174	***	0.432656	***	0.708677	***	0.7124	***
Natural killer cell (NK)	XCL1	0.3917	***	0.398406	***	0.306587	***	0.308769	***
	KIR3DL1	0.0478	3.58E-01	0.030733	5.69E-01	-0.04713	2.85E-01	-0.0550	2.30E-01
	CD7	0.4936	***	0.507592	***	0.698096	***	0.7086	***
Neutrophil	CD15	0.5278	***	0.507572	***	0.564066	***	0.5500	***
	MPO	0.2179	***	0.19976	***	-0.23984	***	-0.2301	***
Dendritic cell (DC)	CD1C	0.3222	***	0.314479	***	0.322677	***	0.3310	***
	CD141	0.2562	***	0.249987	***	0.175303	***	0.1695	***

None, correlation without adjustment; Purity, correlation adjusted for tumor purity; Cor, R value of Spearman`s correlation. *p < 0.05, **p < 0.01, ***p < 0.001.

### Gene Sets Enriched in *SLC1A5* Expression Phenotype

**Figure 5:**
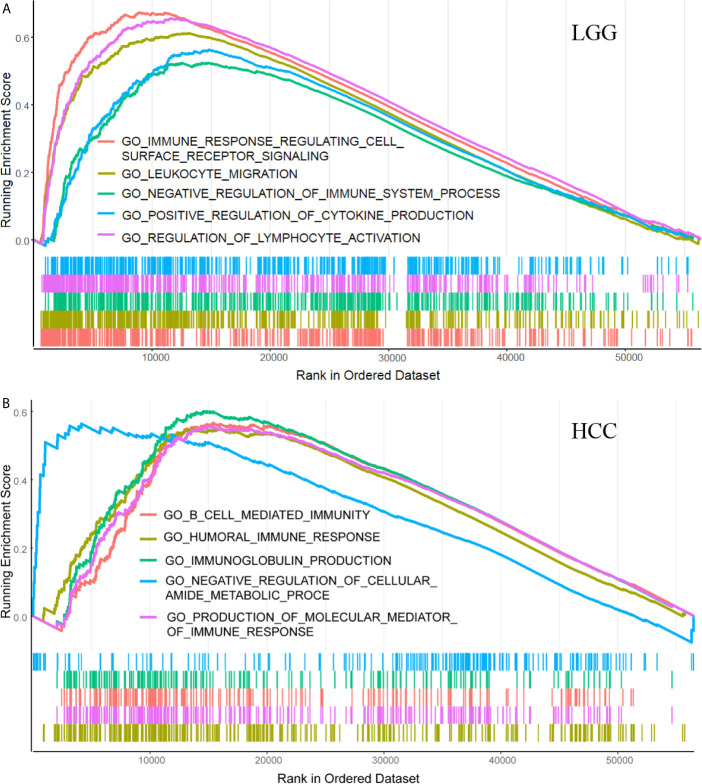
GSEA results showing differential enrichment of genes in GO with high and low *SLC1A5* expression in LGG **(A)** and HCC **(B)**.

GSEA was implemented to reveal differences in enrichment of GO terms in the high *SLC1A5*-expression group. For the sake of simplicity, only the top five GO terms of high expression in LGG and HCC are listed here on the basis of the normalized enrichment score (NES). In LGG, as shown in **Figure 5A**, high *SLC1A5* phenotype sets were enriched in the top five GO items, namely humoral immune response, production of molecular mediator of immune response, B-cell-mediated immunity, immunoglobulin production, and negative regulation of cellular amide metabolic process. In HCC, five significant GO items also were identified in the *SLC1A5* high-expression phenotype, namely immune response-regulating cell-surface receptor signaling pathway, leukocyte migration, negative regulation of immune system process, regulation of lymphocyte activation, and positive regulation of cytokine production (**Figure 5B**). These results indicate the presence of a strong relation between tumor immunity and *SLC1A5* expression in LGG and HCC.

## Discussion

Although *SLC1A5* has not been well investigated for its role in tumor immunity, it is known that *SLC1A5* is required in Th1 and T helper-cell 17 (Th17) induction and is a major regulator of glutamine transport in T lymphocytes (37–39). The current study demonstrated that high *SLC1A5* expression correlated significantly with a poor prognosis in bladder and breast cancers, renal clear-cell carcinoma, LGG, HCC, mesothelioma, and uveal melanoma. We further found that high expression of *SLC1A5* was especially associated with a poor prognosis of HCC in males, Asians, those without hepatitis viral infections, and those having tumors with micro-vascular invasion. Additionally, *SLC1A5* expression correlated significantly with infiltration of multiple immune cells based on the mRNA amounts of markers for different immune cell types in LGG and HCC. Taken together, these findings demonstrated the presence of a strong correlation of *SLC1A5* expression with prognosis and tumor immunology in LGG and HCC.

Glutamine is an essential nutrient regulating energy production, redox homeostasis, and signaling in cancer cells. There also are some glutamine inhibitors with plausible clinical application prospects. SLC1A5, a key transporter in charge of moving glutamine across the cytomembrane and mitochondrial membrane, is involved in several cancers (40, 41). Recently, *SLC1A5* has received great attention for its membrane protein characteristics and potential role in cancers, which has made it a druggable therapeutic target. Schulte et al. (42) discovered the first small molecule inhibitor targeted at SLC1A5 specifically, named V-9302. Blocking the expression of SLC1A5 through V-9302 reduces cancer cell growth and proliferation and increases cell death and oxidative stress, thereby leading to antitumor efficacy both *in vitro* and *in vivo* (39, 42). Other inhibitors of *SLC1A5*, such as 6-diazo-5-oxo-l-norleucine (DON), benzylserine, and L-γ-glutamyl-p-nitroanilide (GPNA), also showed suppression of tumor growth (22, 43).

The expression of *SLC1A5* is upregulated in breast cancer, colon cancer, lung cancer, melanoma, neuroblastoma, glioblastoma, and prostate cancer (40). In this study, we found that *SLC1A5* was overexpressed in most cancer tissues compared with normal ones using independent datasets in Oncomine (see **Figure 1A**). The 17 types of the total 24 tumor types in the TCGA dataset showed significantly higher expression of *SLC1A5* than their normal counterparts (**Figures 1B, C**). Although there exist some variations in different types of cancer, which might be caused by the different data collection criteria used in each study and underlying causative mechanisms, *SLC1A5* generally is upregulated in cancers. These cancers with high *SLC1A5* expression may be dependent on glutamine and respond to glutamine inhibitors, either alone or in combination with other therapies.

Further, we demonstrated the prognostic value of *SLC1A5* in pan-cancer. Prognostic analysis of data from PrognoScan and Kaplan-Meier plotter showed that high *SLC1A5* expression correlated with a poor prognosis in bladder, brain, breast, gastric, and skin cancers. Gene expression analysis of the TCGA dataset revealed that high expression of *SLC1A5* correlated significantly with a poorer prognosis in KIRC, LGG, HCC, MESO, and UVM. Meanwhile, in STAD, high *SLC1A5* correlated with a better prognosis. *SLC1A5* expression shows promising prognostic power, as the AUC for predicting overall survival in LGG and HCC was 0.717 and 0.727, respectively (**Figure 3**). These results strongly highlight the role of upregulated *SLC1A5* as a prognostic factor for poor survival in LGG and HCC. Especially, high *SLC1A5* expression might impact the prognosis of HCC patients with the following characteristics: male, stage 3, grade 2, AJCC_T3, Asian, and micro-vascular invasion. One possible mechanism for the explanation of the correlation detected between *SLC1A5* expression and poor prognosis might be that *SLC1A5* contributes to cancer cell uptake of glutamine, which led to activation of the mTORC1 pathway as well as promotion of cancer cell proliferation. Besides, a recently reported novel splice variant of *SLC1A5* is a mitochondrial glutamine transporter, which is critical for metabolic reprogramming of cancer cells, thus supporting carcinogenesis (27). Paradoxically, Bothwell et al. have shown that *SLC1A5* knockout in human epithelial and mesenchymal HCC cell lines failed to affect cellular proliferation or the mTORCA pathway (26). These results imply that *SLC1A5* is nonessential for these cultured cell lines. However, our results clearly indicate that *SLC1A5* can serve as a prognostic biomarker in diverse cancers, including HCC.

Another important aspect of this study is that *SLC1A5* expression correlated with the extents of multiple immune cell infiltration in cancers, especially LGG and HCC. As shown in **Figure 4**, our study with the TIMER database demonstrated that *SLC1A5* expression had a moderate to strong correlation with all six immune cell-type infiltrations in LGG and HCC, except for CD8^+^ T cells in LGG. The TME is a complex milieu of non-cancerous cells consisting mainly of immune cells around tumor cells. Genes highly expressed in TME cells are believed to have negative associations with tumor purity. *SLC1A5* expression in both LGG and HCC showed significantly negative correlations with their tumor purities, indicating its comparative expression in the TME. Moreover, the correlation between *SLC1A5* expression and the marker genes of immune cells implicate *SLC1A5* in regulating tumor immunology in LGG and HCC. In both of these cancers, most of the 48 marker genes showed a positive correlation with *SLC1A5* expression, with the highest correlation being found between *SLC1A5* and a T helper cell 9 (Th9) marker *PU.1* (*SPI1*), which is an oncogene (44). A previous study suggested that Th9 cells play a tumor-promoting role in HCC, and a high infiltrating Th9 number reflects worse survival (45). Additionally, a weak to moderate correlation was found between *SLC1A5* expression and the regulation of several markers of other T helper cells (Th1, Th2, Th17, Th22) in LGG and HCC. It has been shown that Th1 cells were significantly associated with a good prognosis in patients with HCC, whereas Th2, TH17, and TH22 cells were related to tumor growth, metastasis, and poor clinical outcomes in HCC (46–49). Furthermore, increased *SLC1A5* expression was positively correlated with the expression of regulatory T cells (Tregs) and the induction of T-cell exhaustion markers (CCR8, CD25, PD-1, and CTLA4 in HCC and LGG). Regulatory T cells (Tregs) suppress T-cell immunity in HCC and are associated with a bad prognosis (50) and T-cell exhaustion leading to immune escape represents one of the mechanisms for cancer cells to get rid of control from the immune system (51). CD8^+^ T-cell markers showed a significantly positive correlation with *SLC1A5* expression in HCC. The general consensus is that tumors with CD8^+^ T cell infiltration could be triggered to some level of antitumor immunity by anti–PD-1 therapies (52). Previous research has showed that in *SLC1A5*-deficient T cells, glutamine uptake and mTORC1 activation on TCR engagement were largely impaired, and the polarization of Th1 and Th17 were also blocked (53). This might in part explain the widely positive correlations between *SLC1A5* and T cells (54). Moreover, the *SLC1A5* expression was found to be positively correlated with several gene markers for monocyte, macrophage, natural killer cell (NK), neutrophil, and DC. Monocyte and macrophage can induce NK cell dysfunction in advanced-stage HCC (55). While DC was found with at least three subtypes, DC vaccine could improve PD-1 inhibitor therapeutic effect in a HCC mouse model (56). Our findings suggest that *SLC1A5* correlates positively with these immune cells, while the specific biological functions and processes need to be further explored. Furthermore, we searched for GO term enriched in *SLC1A5*-overexpressing datasets and found that upregulated *SLC1A5* expression was primarily linked with immune cell and immune process in LGG and HCC. These results support the idea that high expression of *SLC1A5* in patients with LGG and HCC could change their tumor immunology, which may eventually influence patient survival. Previous studies have provided possible mechanisms that can explain why *SLC1A5* expression correlates positively with immune cell infiltration and a poor prognosis. Glutamine, as an immunomodulatory nutrient and fuel for tumor cells, is transported by *SLC1A5*. In addition, the consequence of glutamine metabolism in cancer cells is the creation of a hypoxic, acidic, and nutrient-depleted tumor microenvironment (TME) (19, 57). The tumor immune microenvironment in HCC has been characterized as shifted from help resisting tumors toward immunosuppression (58). The second-highest correlation was found between *SLC1A5* expression and *CD86*, a marker gene of tumor-associated macrophages with the ability to capture PD1-targeting antibodies on its surface and consequently limiting immunotherapeutic efficacy (59). In particular, the expression of *SLC1A5* correlates significantly with the prototypical immunotherapeutic gene biomarkers, including *PD-1* and *CTLA4*. These correlations collectively indicate a potential role for *SLC1A5* expression as a biomarker to predict a clinical benefit of using immune-checkpoint inhibitors to treat cancers, especially LGG and HCC. Future investigation is needed to realize the definitive clinical value of *SLC1A5* as predictive markers in immunotherapy.

Powell and colleagues reported that combining JHU83 with anti-PD1 antibody can dramatically improve anti-PD1 antitumor effects compared with anti-PD1 therapy alone (19, 60). Two Phase II clinical trials have been conducted that combined glutamine and a glutamate pathway inhibitor with immune-checkpoint inhibitors. As a potential target in abnormal metabolism of glutamine, *SLC1A5* has an outstanding significance for combination therapy studies.

Even though we integrated data across multiple databases, this study had a few limitations. First, the correlation of *SLC1A5* expression and immunity in cancer was analyzed on the basis of publicly available expression datasets. Thus, some experiments need to be performed *in vivo/in vitro*, and single-cell RNA sequencing should be carried out to clarify the different role of *SLC1A5* in immune and cancer cells. Second, despite the finding that *SLC1A5* expression correlates significantly with immune cell infiltration in LGG and HCC, there was no database reflecting the correlation of *SLC1A5* expression and immunotherapeutic response in these cancer types. Future clinical trials focusing on *SLC1A5* expression and immunotherapeutic response in patients with LGG or HCC can help reach a direct conclusion. Finally, combining Leone’s report (19) and our results, we think that it is necessary to investigate the clinical value of a combined SLC1A5 inhibitor (or glutamine inhibitor, alternatively) and an immune-checkpoint inhibitor for treating LGG or HCC patients.

In summary, our results demonstrate that *SLC1A5* is a powerful independent prognostic biomarker for multiple cancers. Specially for LGG and HCC, *SLC1A5* can be used to evaluate the extent of immune cell infiltration in the tumor tissues. On the basis of these findings, we suggest further research on the subject to improve understanding of the immunotherapeutic impact of *SLC1A5*.

## Data Availability Statement

The original contributions presented in the study are included in the article/**Supplementary Material**. Further inquiries can be directed to the corresponding authors.

## Author Contributions

JZ, ZY, MT, WM, and HG participated in literature search and data collection. JZ and ZY conducted the data analysis. JZ, LL, and MDL participated in writing of the manuscript. MDL conceived the study and was involved in every step of the work. All authors contributed to the article and approved the submitted version.

## Funding

This study was supported by the National Science and Technology Major Project of China (2018ZX10302206-001-009), the China Precision Medicine Initiative (2016YFC0906300), Major Program of National Natural Science Foundation of China (81790634), Research Center for Air Pollution and Health of Zhejiang University, and State Key Laboratory for Diagnosis and Treatment of Infectious Diseases of the First Affiliated Hospital of Zhejiang University.

## Conflict of Interest

The authors declare that the research was conducted in the absence of any commercial or financial relationships that could be construed as a potential conflict of interest.
