# Supplementary material for: Correlation Between Prognostic Biomarker SLC1A5 and Immune Infiltrates in Various Types of Cancers Including Hepatocellular Carcinoma
Source: Front Oncol. 2021 Jul 22;11:608641. doi: 10.3389/fonc.2021.608641 (PMC8339971; doi:10.3389/fonc.2021.608641)
Supplement: Supplementary file 1 [file Presentation_1.pptx]

## Slide 1
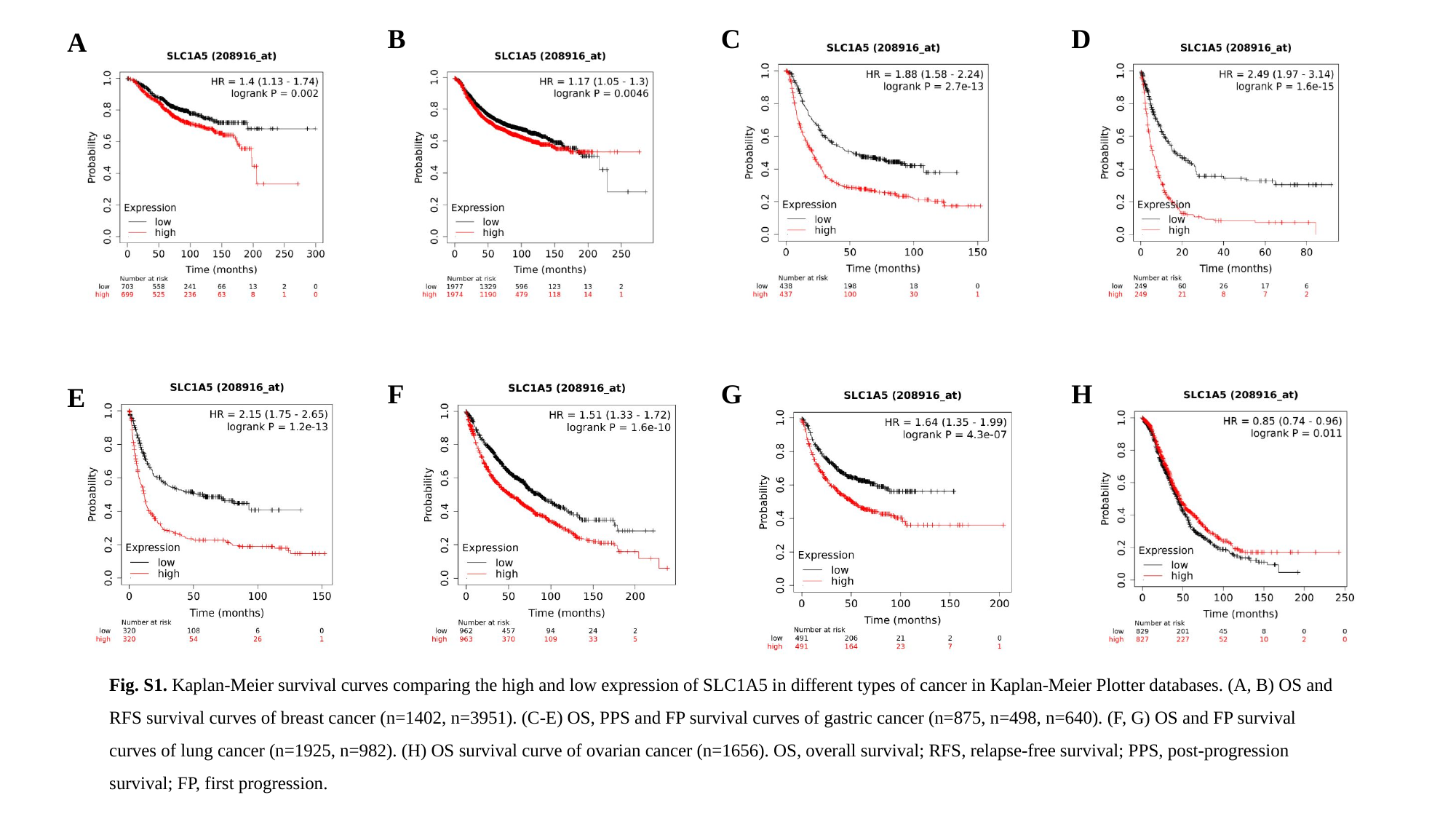

B
C
D
A
F
G
H
E
Fig. S1. Kaplan-Meier survival curves comparing the high and low expression of SLC1A5 in different types of cancer in Kaplan-Meier Plotter databases. (A, B) OS and RFS survival curves of breast cancer (n=1402, n=3951). (C-E) OS, PPS and FP survival curves of gastric cancer (n=875, n=498, n=640). (F, G) OS and FP survival curves of lung cancer (n=1925, n=982). (H) OS survival curve of ovarian cancer (n=1656). OS, overall survival; RFS, relapse-free survival; PPS, post-progression survival; FP, first progression.
