# Supplementary material for: Correlation Between Prognostic Biomarker SLC1A5 and Immune Infiltrates in Various Types of Cancers Including Hepatocellular Carcinoma
Source: Front Oncol. 2021 Jul 22;11:608641. doi: 10.3389/fonc.2021.608641 (PMC8339971; doi:10.3389/fonc.2021.608641)
Supplement: Supplementary file 2 [file Table_1.docx]

**Supplementary Information**

Table S1: *SLC1A5* expression in cancers vs. normal tissue in Oncomine database

| Cancer | Cancer type | p-value | Fold change | Rank (%) | Sample | Reference (PMID) |
| --- | --- | --- | --- | --- | --- | --- |
| Bladder | Superficial Bladder Cancer | 0.000097 | 1.549 | 28% | 157 | J Clin Oncol 2006/02/10 |
| Brain | Glioblastoma | 0.000129 | 2.538 | 9% | 54 | Cancer Res 2005/10/01 |
|  | Anaplastic Astrocytoma | 0.000863 | 1.514 | 15% | 180 | Cancer Cell 2006/04/01 |
|  | Glioblastoma | 2.48e-05 | 1.612 | 19% | 180 | Cancer Cell 2006/04/01 |
| Breast | Lobular Breast Carcinoma | 1.35e-06 | 4.471 | 2% | 64 | Mol Biol Cell 2004/06/01 |
|  | Invasive Ductal Breast Carcinoma | 5.34e-08 | 1.757 | 3% | 64 | Mol Biol Cell 2004/06/01 |
| Myeloma | Multiple Myeloma | 6.64e-20 | 2.686 | 1% | 131 | Blood 2002/03/01 |
|  | Monoclonal Gammopathy of Undetermined Significance | 1.18e-07 | 1.951 | 2% | 78 | Blood 2007/02/15 |
|  | Smoldering Myeloma | 4.51e-05 | 2.835 | 17% | 78 | Blood 2007/02/15 |
| Lymphoma | Burkitt's Lymphoma | 8.40e-11 | 2.42 | 1% | 336 | Nat Genet 2005/04/01 |
|  | Diffuse Large B-Cell Lymphoma | 6.25e-09 | 2.058 | 1% | 336 | Nat Genet 2005/04/01 |
|  | Primary Effusion Lymphoma | 1.26e-06 | 2.722 | 3% | 336 | Nat Genet 2005/04/01 |
|  | Hairy Cell Leukemia | 3.53e-05 | 1.949 | 12% | 336 | Nat Genet 2005/04/01 |
|  | Activated B-Cell-Like Diffuse Large B-Cell Lymphoma | 5.68e-10 | 1.806 | 4% | 136 | Nature 2009/06/04 |
|  | Anaplastic Large Cell Lymphoma | 1.92e-04 | 2.078 | 13% | 60 | J Clin Invest 2007/03/01 |
| Lung | Squamous Cell Lung Carcinoma | 3.33e-11 | 1.929 | 2% | 93 | Cancer Res 2005/04/15 |
|  | Lung Adenocarcinoma | 3.14e-05 | 1.586 | 7% | 39 | Am J Pathol 2005/12/01 |
|  | Lung Adenocarcinoma | 3.96e-10 | 1.688 | 8% | 116 | Genome Res 2012/07/01 |
|  | Squamous Cell Lung Carcinoma | 2.61e-06 | 1.571 | 13% | 156 | PLoS One 2010/04/22 |
| Melanoma | Cutaneous Melanoma | 2.55e-10 | 4.471 | 2% | 70 | Clin Cancer Res 2005/10/15 |
| Colorectal | Colon Adenocarcinoma | 1.24e-13 | 2.731 | 3% | 123 | Int J Cancer 2007/11/01 |
|  | Colorectal Carcinoma | 7.29e-08 | 2.053 | 10% | 82 | Clin Exp Metastasis 2010/02/01 |
|  | Colon Carcinoma | 1.71e-06 | 1.85 | 10% | 40 | PLoS One 2010/10/01 |
|  | Rectal Adenocarcinoma | 1.16e-09 | 1.63 | 13% | 237 | TCGA |
| Head-Neck | Oral Cavity Squamous Cell Carcinoma | 3.22e-13 | 1.786 | 3% | 79 | PLoS One 2011/08/11 |
|  | Tongue Squamous Cell Carcinoma | 7.43e-06 | 2.221 | 11% | 58 | BMC Cancer 2009/01/12 |
| Prostate | Prostate Carcinoma | 3.24e-04 | 2.106 | 6% | 102 | Cancer Cell 2002/03/01 |
|  | Prostate Adenocarcinoma | 5.11e-04 | 1.745 | 4% | 89 | Cancer Res 2008/02/01 |
| Leukemia | Chronic Lymphocytic Leukemia | 6.11e-05 | 2.565 | 7% | 111 | J Clin Oncol 2004/10/01 |
|  | B-Cell Acute Lymphoblastic Leukemia | 3.53e-06 | 1.842 | 14% | 127 | Leukemia 2007/06/01 |
| Esophagus | Esophageal Adenocarcinoma | 3.91e-04 | 3.463 | 8% | 48 | Gastroenterology 2006/09/01 |
| Gastric | Diffuse Gastric Adenocarcinoma | 1.66e-04 | 1.765 | 7% | 90 | Clin Cancer Res 2011/04/01 |
|  | Gastric Intestinal Type Adenocarcinoma | 1.48e-06 | 2.021 | 12% | 69 | Eur J Cancer 2009/02/01 |

Table S2. Significant relation between *SLC1A5* expression and patient prognosis of different cancers in PrognoScan database

| Dataset | Cancer Type | Subtype | Endpoint | COX p-value | HR [95% CI] |
| --- | --- | --- | --- | --- | --- |
| GSE13507 | Bladder cancer |  | Overall Survival | 0.031506 | 1.51 [1.04 -2.19] |
| GSE16131-GPL96 | Blood cancer | Follicular lymphoma | Overall Survival | 0.031646 | 1.69 [1.05 -2.73] |
| GSE4271-GPL96 | Brain cancer | Astrocytoma | Overall Survival | 0.006876 | 1.96 [1.20 -3.19] |
| GSE19615 | Breast cancer |  | Distant Metastasis Free Survival | 0.017113 | 3.36 [1.24-9.10] |
| GSE9893 | Breast cancer |  | Overall Survival | 0.000589 | 1.39 [1.15-1.68] |
| GSE2034 | Breast cancer |  | Distant Metastasis Free Survival | 0.036301 | 0.64 [0.42-0.97] |
| GSE1456-GPL96 | Breast cancer |  | Relapse Free Survival | 0.02541 | 2.21 [1.10-4.42] |
| GSE1456-GPL96 | Breast cancer |  | Disease Specific Survival | 0.012905 | 2.77 [1.24-6.18] |
| GSE3494-GPL96 | Breast cancer |  | Disease Specific Survival | 0.029513 | 2.16 [1.08-4.32] |
| GSE9891 | Ovarian cancer |  | Overall Survival | 0.018125 | 0.66 [0.47-0.93] |
| GSE19234 | Skin cancer | Melanoma | Overall Survival | 0.012235 | 3.87 [1.34-11.14] |

Table S3 Correlation of *SLC1A5* expression with immune infiltration level in diverse type cancers via TIMER database.

| Cancer type | Variable | Partial Corr | p |
| --- | --- | --- | --- |
| ACC | Purity | -0.1877 | 0.109238 |
| ACC | B Cell | 0.1901 | 0.107293 |
| ACC | CD8+ T Cell | 0.2604 | 0.026092 |
| ACC | CD4+ T Cell | 0.3699 | 0.001278 |
| ACC | Macrophage | 0.1070 | 0.367729 |
| ACC | Neutrophil | 0.4103 | 0.000311 |
| ACC | Dendritic Cell | 0.4975 | 7.52e-06 |
| BLCA | Purity | -0.0574 | 0.271599 |
| BLCA | B Cell | 0.0516 | 0.326683 |
| BLCA | CD8+ T Cell | 0.1309 | 0.012202 |
| BLCA | CD4+ T Cell | -0.1393 | 0.007711 |
| BLCA | Macrophage | 0.1918 | 0.000229 |
| BLCA | Neutrophil | 0.0097 | 0.854439 |
| BLCA | Dendritic Cell | 0.0067 | 0.898419 |
| BRCA | Purity | 0.1377 | 1.31e-05 |
| BRCA | B Cell | 0.0124 | 0.699575 |
| BRCA | CD8+ T Cell | -0.0907 | 0.00459 |
| BRCA | CD4+ T Cell | -0.0559 | 0.083008 |
| BRCA | Macrophage | -0.0932 | 0.003462 |
| BRCA | Neutrophil | -0.0238 | 0.464052 |
| BRCA | Dendritic Cell | -0.0176 | 0.587261 |
| BRCA-Basal | Purity | 0.1386 | 0.117143 |
| BRCA-Basal | B Cell | 0.1060 | 0.241497 |
| BRCA-Basal | CD8+ T Cell | -0.1281 | 0.157924 |
| BRCA-Basal | CD4+ T Cell | 0.0506 | 0.580219 |
| BRCA-Basal | Macrophage | -0.1339 | 0.133323 |
| BRCA-Basal | Neutrophil | -0.0437 | 0.650664 |
| BRCA-Basal | Dendritic Cell | -0.0461 | 0.626442 |
| BRCA-Her2 | Purity | -0.0229 | 0.86354 |
| BRCA-Her2 | B Cell | -0.0141 | 0.916553 |
| BRCA-Her2 | CD8+ T Cell | -0.0974 | 0.47122 |
| BRCA-Her2 | CD4+ T Cell | -0.3030 | 0.020765 |
| BRCA-Her2 | Macrophage | -0.1717 | 0.197623 |
| BRCA-Her2 | Neutrophil | -0.2909 | 0.026731 |
| BRCA-Her2 | Dendritic Cell | -0.2234 | 0.097914 |
| BRCA-Luminal | Purity | 0.1592 | 0.000187 |
| BRCA-Luminal | B Cell | -0.0327 | 0.447372 |
| BRCA-Luminal | CD8+ T Cell | -0.1614 | 0.000172 |
| BRCA-Luminal | CD4+ T Cell | -0.0753 | 0.081453 |
| BRCA-Luminal | Macrophage | -0.0999 | 0.019973 |
| BRCA-Luminal | Neutrophil | -0.0524 | 0.226624 |
| BRCA-Luminal | Dendritic Cell | -0.0390 | 0.368431 |
| CESC | Purity | -0.0302 | 0.616406 |
| CESC | B Cell | -0.0714 | 0.236575 |
| CESC | CD8+ T Cell | -0.0010 | 0.986594 |
| CESC | CD4+ T Cell | 0.0241 | 0.689235 |
| CESC | Macrophage | -0.1260 | 0.036122 |
| CESC | Neutrophil | 0.1007 | 0.094275 |
| CESC | Dendritic Cell | 0.0456 | 0.450473 |
| CHOL | Purity | -0.0403 | 0.81551 |
| CHOL | B Cell | 0.1360 | 0.435854 |
| CHOL | CD8+ T Cell | 0.0731 | 0.67654 |
| CHOL | CD4+ T Cell | 0.1611 | 0.355315 |
| CHOL | Macrophage | 0.1336 | 0.444112 |
| CHOL | Neutrophil | 0.3357 | 0.04866 |
| CHOL | Dendritic Cell | 0.2219 | 0.200098 |
| COAD | Purity | 0.0882 | 0.07543 |
| COAD | B Cell | -0.2916 | 2.32e-09 |
| COAD | CD8+ T Cell | -0.1766 | 0.000351 |
| COAD | CD4+ T Cell | 0.0093 | 0.852568 |
| COAD | Macrophage | -0.0604 | 0.225429 |
| COAD | Neutrophil | -0.1186 | 0.017549 |
| COAD | Dendritic Cell | -0.1623 | 0.001091 |
| DLBC | Purity | -0.0555 | 0.726893 |
| DLBC | B Cell | 0.0445 | 0.860902 |
| DLBC | CD8+ T Cell | -0.2362 | 0.302572 |
| DLBC | CD4+ T Cell | -0.2001 | 0.384441 |
| DLBC | Macrophage | -0.1799 | 0.435104 |
| DLBC | Neutrophil | 0.2396 | 0.295491 |
| DLBC | Dendritic Cell | 0.3961 | 0.075491 |
| ESCA | Purity | 0.2430 | 0.000981 |
| ESCA | B Cell | -0.0018 | 0.98074 |
| ESCA | CD8+ T Cell | -0.1361 | 0.068574 |
| ESCA | CD4+ T Cell | 0.0162 | 0.829133 |
| ESCA | Macrophage | -0.0309 | 0.680594 |
| ESCA | Neutrophil | -0.2030 | 0.006276 |
| ESCA | Dendritic Cell | -0.2400 | 0.001174 |
| GBM | Purity | -0.1256 | 0.010086 |
| GBM | B Cell | -0.1138 | 0.019959 |
| GBM | CD8+ T Cell | -0.2384 | 8.16e-07 |
| GBM | CD4+ T Cell | -0.0431 | 0.379316 |
| GBM | Macrophage | -0.0012 | 0.980939 |
| GBM | Neutrophil | -0.0286 | 0.560179 |
| GBM | Dendritic Cell | 0.2630 | 4.83e-08 |
| HNSC | Purity | 0.1655 | 0.000224 |
| HNSC | B Cell | 0.0561 | 0.221441 |
| HNSC | CD8+ T Cell | 0.0474 | 0.303186 |
| HNSC | CD4+ T Cell | 0.1755 | 0.000111 |
| HNSC | Macrophage | 0.1335 | 0.003294 |
| HNSC | Neutrophil | 0.1138 | 0.012727 |
| HNSC | Dendritic Cell | 0.0982 | 0.031049 |
| HNSC-HPVpos | Purity | 0.0195 | 0.855117 |
| HNSC-HPVpos | B Cell | 0.2349 | 0.037185 |
| HNSC-HPVpos | CD8+ T Cell | 0.1903 | 0.095096 |
| HNSC-HPVpos | CD4+ T Cell | 0.2040 | 0.066017 |
| HNSC-HPVpos | Macrophage | 0.0717 | 0.511882 |
| HNSC-HPVpos | Neutrophil | 0.2612 | 0.015758 |
| HNSC-HPVpos | Dendritic Cell | 0.1834 | 0.097067 |
| HNSC-HPVneg | Purity | 0.1640 | 0.000981 |
| HNSC-HPVneg | B Cell | -0.0222 | 0.660634 |
| HNSC-HPVneg | CD8+ T Cell | -0.0025 | 0.960312 |
| HNSC-HPVneg | CD4+ T Cell | 0.1621 | 0.001229 |
| HNSC-HPVneg | Macrophage | 0.1347 | 0.007433 |
| HNSC-HPVneg | Neutrophil | 0.0722 | 0.153999 |
| HNSC-HPVneg | Dendritic Cell | 0.0661 | 0.189418 |
| KICH | Purity | -0.0779 | 0.534027 |
| KICH | B Cell | 0.1380 | 0.273028 |
| KICH | CD8+ T Cell | -0.0036 | 0.977136 |
| KICH | CD4+ T Cell | 0.1846 | 0.140971 |
| KICH | Macrophage | -0.0712 | 0.572806 |
| KICH | Neutrophil | 0.3360 | 0.006207 |
| KICH | Dendritic Cell | 0.2695 | 0.029952 |
| KIRC | Purity | -0.0005 | 0.990841 |
| KIRC | B Cell | 0.0879 | 0.060023 |
| KIRC | CD8+ T Cell | 0.1245 | 0.009117 |
| KIRC | CD4+ T Cell | -0.0122 | 0.793497 |
| KIRC | Macrophage | -0.0095 | 0.841818 |
| KIRC | Neutrophil | 0.1272 | 0.006434 |
| KIRC | Dendritic Cell | 0.1670 | 0.000347 |
| KIRP | Purity | 0.0394 | 0.527768 |
| KIRP | B Cell | 0.0978 | 0.118662 |
| KIRP | CD8+ T Cell | 0.0497 | 0.426524 |
| KIRP | CD4+ T Cell | -0.0243 | 0.697834 |
| KIRP | Macrophage | 0.1407 | 0.026425 |
| KIRP | Neutrophil | -0.0415 | 0.506469 |
| KIRP | Dendritic Cell | -0.0170 | 0.786253 |
| LGG | Purity | -0.3438 | 9.82e-15 |
| LGG | B Cell | 0.5564 | 3.29E-40 |
| LGG | CD8+ T Cell | 0.0828 | 0.070626 |
| LGG | CD4+ T Cell | 0.8000 | 3.35e-107 |
| LGG | Macrophage | 0.7294 | 1.10e-79 |
| LGG | Neutrophil | 0.7595 | 2.23e-90 |
| LGG | Dendritic Cell | 0.7562 | 2.48e-89 |
| LIHC (HCC) | Purity | -0.2771 | 1.62e-07 |
| LIHC (HCC) | B Cell | 0.3991 | 1.38e-14 |
| LIHC (HCC) | CD8+ T Cell | 0.3947 | 3.39e-14 |
| LIHC (HCC) | CD4+ T Cell | 0.4066 | 3.94e-15 |
| LIHC (HCC) | Macrophage | 0.5669 | 2.17e-30 |
| LIHC (HCC) | Neutrophil | 0.4381 | 1.29e-17 |
| LIHC (HCC) | Dendritic Cell | 0.5101 | 6.45e-24 |
| LUAD | Purity | 0.0988 | 0.02817 |
| LUAD | B Cell | -0.0813 | 0.073867 |
| LUAD | CD8+ T Cell | -0.0770 | 0.089747 |
| LUAD | CD4+ T Cell | -0.0808 | 0.075814 |
| LUAD | Macrophage | -0.2034 | 6.36e-06 |
| LUAD | Neutrophil | -0.1666 | 0.000235 |
| LUAD | Dendritic Cell | -0.1916 | 2.03e-05 |
| LUSC | Purity | 0.1158 | 0.011255 |
| LUSC | B Cell | -0.1959 | 1.81e-05 |
| LUSC | CD8+ T Cell | -0.0946 | 0.039324 |
| LUSC | CD4+ T Cell | -0.1930 | 2.29e-05 |
| LUSC | Macrophage | -0.0720 | 0.116292 |
| LUSC | Neutrophil | -0.2038 | 7.43e-06 |
| LUSC | Dendritic Cell | -0.2047 | 7.04e-06 |
| MESO | Purity | -0.2546 | 0.018001 |
| MESO | B Cell | 0.0988 | 0.371095 |
| MESO | CD8+ T Cell | 0.1245 | 0.259354 |
| MESO | CD4+ T Cell | 0.2366 | 0.030245 |
| MESO | Macrophage | 0.2243 | 0.040244 |
| MESO | Neutrophil | -0.1381 | 0.210474 |
| MESO | Dendritic Cell | 0.3193 | 0.003067 |
| OV | Purity | -0.1040 | 0.022112 |
| OV | B Cell | 0.0324 | 0.479285 |
| OV | CD8+ T Cell | 0.0446 | 0.329464 |
| OV | CD4+ T Cell | 0.0052 | 0.909146 |
| OV | Macrophage | -0.0865 | 0.058205 |
| OV | Neutrophil | 0.0588 | 0.198555 |
| OV | Dendritic Cell | 0.0439 | 0.337573 |
| PAAD | Purity | 0.0352 | 0.646426 |
| PAAD | B Cell | 0.1014 | 0.186849 |
| PAAD | CD8+ T Cell | 0.1489 | 0.051986 |
| PAAD | CD4+ T Cell | -0.2316 | 0.002452 |
| PAAD | Macrophage | -0.0487 | 0.527499 |
| PAAD | Neutrophil | 0.0579 | 0.452269 |
| PAAD | Dendritic Cell | 0.1270 | 0.097818 |
| PCPG | Purity | -0.1759 | 0.022583 |
| PCPG | B Cell | 0.3677 | 1.01e-06 |
| PCPG | CD8+ T Cell | 0.0400 | 0.607566 |
| PCPG | CD4+ T Cell | 0.1701 | 0.027935 |
| PCPG | Macrophage | 0.1597 | 0.039895 |
| PCPG | Neutrophil | 0.1457 | 0.060225 |
| PCPG | Dendritic Cell | 0.3732 | 6.78e-07 |
| PRAD | Purity | -0.1258 | 0.010142 |
| PRAD | B Cell | -0.1098 | 0.025889 |
| PRAD | CD8+ T Cell | 0.1924 | 7.86e-05 |
| PRAD | CD4+ T Cell | -0.0538 | 0.276704 |
| PRAD | Macrophage | 0.1190 | 0.015133 |
| PRAD | Neutrophil | -0.0147 | 0.765902 |
| PRAD | Dendritic Cell | 0.0126 | 0.798544 |
| READ | Purity | 0.1675 | 0.047925 |
| READ | B Cell | -0.1016 | 0.233837 |
| READ | CD8+ T Cell | -0.1275 | 0.134616 |
| READ | CD4+ T Cell | -0.0008 | 0.992252 |
| READ | Macrophage | 0.0256 | 0.764694 |
| READ | Neutrophil | -0.1496 | 0.079837 |
| READ | Dendritic Cell | -0.1126 | 0.186869 |
| SARC | Purity | -0.2829 | 6.89e-06 |
| SARC | B Cell | 0.0664 | 0.306521 |
| SARC | CD8+ T Cell | 0.0238 | 0.713238 |
| SARC | CD4+ T Cell | 0.1534 | 0.017667 |
| SARC | Macrophage | 0.4185 | 2.21e-11 |
| SARC | Neutrophil | 0.1868 | 0.003536 |
| SARC | Dendritic Cell | 0.3505 | 2.26e-08 |
| SKCM | Purity | 0.0568 | 0.225435 |
| SKCM | B Cell | 0.0639 | 0.176803 |
| SKCM | CD8+ T Cell | 0.0150 | 0.753615 |
| SKCM | CD4+ T Cell | 0.0555 | 0.241769 |
| SKCM | Macrophage | 0.0797 | 0.090356 |
| SKCM | Neutrophil | 0.0579 | 0.218949 |
| SKCM | Dendritic Cell | 0.1693 | 0.00033 |
| SKCM-Primary | Purity | 0.1163 | 0.241952 |
| SKCM-Primary | B Cell | 0.0894 | 0.373836 |
| SKCM-Primary | CD8+ T Cell | -0.0107 | 0.91567 |
| SKCM-Primary | CD4+ T Cell | -0.0992 | 0.323693 |
| SKCM-Primary | Macrophage | 0.1358 | 0.175558 |
| SKCM-Primary | Neutrophil | 0.0427 | 0.672923 |
| SKCM-Primary | Dendritic Cell | 0.1910 | 0.055742 |
| SKCM-Metastasis | Purity | 0.0375 | 0.480822 |
| SKCM-Metastasis | B Cell | 0.0021 | 0.968502 |
| SKCM-Metastasis | CD8+ T Cell | -0.0290 | 0.597009 |
| SKCM-Metastasis | CD4+ T Cell | 0.0457 | 0.398401 |
| SKCM-Metastasis | Macrophage | 0.0129 | 0.810238 |
| SKCM-Metastasis | Neutrophil | -0.0233 | 0.663622 |
| SKCM-Metastasis | Dendritic Cell | 0.0937 | 0.082833 |
| STAD | Purity | 0.0866 | 0.091768 |
| STAD | B Cell | -0.1853 | 0.000347 |
| STAD | CD8+ T Cell | -0.1859 | 0.000324 |
| STAD | CD4+ T Cell | -0.2627 | 3.43e-07 |
| STAD | Macrophage | -0.3608 | 8.14e-13 |
| STAD | Neutrophil | -0.1881 | 0.000268 |
| STAD | Dendritic Cell | -0.2305 | 7.25e-06 |
| TGCT | Purity | 0.2347 | 0.004094 |
| TGCT | B Cell | -0.1668 | 0.043531 |
| TGCT | CD8+ T Cell | -0.1152 | 0.164612 |
| TGCT | CD4+ T Cell | 0.0728 | 0.382666 |
| TGCT | Macrophage | 0.1102 | 0.184112 |
| TGCT | Neutrophil | 0.1501 | 0.069656 |
| TGCT | Dendritic Cell | -0.0701 | 0.400451 |
| THCA | Purity | -0.1205 | 0.007648 |
| THCA | B Cell | 0.3571 | 6.03e-16 |
| THCA | CD8+ T Cell | 0.1604 | 0.00038 |
| THCA | CD4+ T Cell | 0.2567 | 8.77e-09 |
| THCA | Macrophage | 0.1896 | 2.48e-05 |
| THCA | Neutrophil | 0.4426 | 7.91e-25 |
| THCA | Dendritic Cell | 0.4917 | 6.81e-31 |
| THYM | Purity | -0.0952 | 0.309167 |
| THYM | B Cell | 0.4717 | 1.18e-07 |
| THYM | CD8+ T Cell | 0.1652 | 0.079041 |
| THYM | CD4+ T Cell | 0.3392 | 0.000271 |
| THYM | Macrophage | 0.3615 | 7.77e-05 |
| THYM | Neutrophil | -0.0782 | 0.408352 |
| THYM | Dendritic Cell | 0.4701 | 1.31e-07 |
| UCEC | Purity | 0.1316 | 0.024047 |
| UCEC | B Cell | 0.1465 | 0.012646 |
| UCEC | CD8+ T Cell | 0.0721 | 0.221801 |
| UCEC | CD4+ T Cell | 0.0929 | 0.113895 |
| UCEC | Macrophage | -0.0156 | 0.791382 |
| UCEC | Neutrophil | 0.1012 | 0.083826 |
| UCEC | Dendritic Cell | 0.1570 | 0.007172 |
| UCS | Purity | -0.1016 | 0.464581 |
| UCS | B Cell | 0.0711 | 0.612687 |
| UCS | CD8+ T Cell | 0.0547 | 0.697208 |
| UCS | CD4+ T Cell | -0.1025 | 0.465109 |
| UCS | Macrophage | 0.0580 | 0.679868 |
| UCS | Neutrophil | 0.1215 | 0.385987 |
| UCS | Dendritic Cell | -0.0412 | 0.769852 |
| UVM | Purity | 0.3127 | 0.005319 |
| UVM | B Cell | -0.2512 | 0.02969 |
| UVM | CD8+ T Cell | 0.1412 | 0.220578 |
| UVM | CD4+ T Cell | -0.0501 | 0.667151 |
| UVM | Macrophage | -0.0710 | 0.577136 |
| UVM | Neutrophil | -0.4577 | 2.86e-05 |
| UVM | Dendritic Cell | 0.3380 | 0.003227 |
